# Echocardiographic markers of inducible myocardial ischemia at baseline evaluation preparatory to exercise stress echocardiography

**DOI:** 10.1186/s12947-016-0064-5

**Published:** 2016-06-01

**Authors:** Antonella Cherubini, Giovanni Cioffi, Carmine Mazzone, Giorgio Faganello, Giulia Barbati, Luigi Tarantini, Giulia Russo, Carlo Stefenelli, Franco Humar, Eliana Grande, Maurizio Fisicaro, Claudio Pandullo, Andrea Di Lenarda

**Affiliations:** 1 Cardiovascular Center, Health Authority n° 1 and University of Trieste, Trieste, Italy; 2 Cardiology Department Villa Bianca Hospital, Trento, Italy; 3 Cardiology Department St. Martino Hospital Azienda Sanitaria Locale n. 1, Belluno, Italy; 4 Echocardiography Laboratory, Villa Bianca Hospital, via Piave 78, 38100 Trento, Italy

**Keywords:** Inducible myocardial ischemia, Exercise stress test, Left ventricular systolic function, Longitudinal function, Cardiovascular risk

## Abstract

**Background:**

Tissue Doppler Imaging (TDI) is a sensible and feasible method to detect longitudinal left ventricular (LV) systolic dysfunction (LVSD) in patients with diabetes mellitus, hypertension or ischemic heart disease. In this study, we hypothesized that longitudinal LVSD assessed by TDI predicted inducible myocardial ischemia independently of other echocardiographic variables (assessed as coexisting potential markers) in patients at increased cardiovascular (CV) risk.

**Methods:**

Two hundred one patients at high CV risk defined according to the ESC Guidelines 2012 underwent exercise stress echocardiography (ExSEcho) for primary prevention. Echocardiographic parameters were measured at rest and peak exercise.

**Results:**

ExSEcho classified 168 (83.6 %) patients as non-ischemic and 33 (16,4 %) as ischemic. Baseline clinical characteristics were similar between the groups, but ischemic had higher blood pressure, received more frequently beta-blockers and antiplatelet agents than non-ischemic patients. The former had greater LV size, lower relative wall thickness and higher left atrial systolic force (LASF) than the latter. LV systolic longitudinal function (measure as peak S’) was significantly lower in ischemic than non-ischemic patients (8.7 ± 2.1 vs 9.7 ± 2.7 cm/sec, *p* = 0.001). The factors independently related to myocardial ischemia at multivariate logistic analysis were: lower peak S’, higher LV circumferential end-systolic stress and LASF.

**Conclusions:**

In asymptomatic patients at increased risk for adverse CV events baseline longitudinal LVSD together with higher LV circumferential end-systolic stress and LASF were the factors associated with myocardial ischemia induced by ExSEcho. The assessment of these factors at standard echocardiography might help the physicians for improving the risk stratification among these patients for ExSEcho.

## Background

Tissue Doppler Imaging (TDI) echocardiography is a sensible and feasible tool to detect subclinical left ventricular (LV) systolic dysfunction (LVSD). In particular, the assessment of mitral annular peak systolic velocity (peak S’) by Tissue-Doppler pulsed wave spectral analysis, reveals the longitudinal LVSD in several settings of patients at increased risk for cardiovascular (CV) adverse events such as those with type 2 diabetes mellitus, arterial hypertension, ischemic heart disease and/or heart failure with preserved LV ejection fraction (LVEF) [1–7]. As a result, peak S’ has emerged as one of the strongest predictors for CV disease in the domain of non-invasive cardiac imaging.

We prospectively studied a large cohort of patients at increased CV risk without history of cardiac disease to test the hypothesis that longitudinal LVSD measured as lower peak S’ at baseline echocardiographic evaluation can predict inducible myocardial ischemia during exercise stress echocardiography (ExSEcho) in these patients. Furthermore, we verified whether other echocardiographic parameters were prognosticators of inducible myocardial ischemia measured at baseline evaluation preceding ExSEcho.

## Methods

### Study population

The study patients were consecutively recruited from the 1^st^ January 2012 to the 31^st^ June 2013 by 2 Italian referral centers (Trieste and Trento) into a prospective evaluation and their data collected in the same database. We selected outpatients aged >18 years without a history/symptoms of cardiac disease who had an increased (high or very high) risk of CV events according to the European Guidelines on Cardiovascular Disease prevention [8]. Patients with a 10-year European SCORE risk > 5 % were considered eligible for this study [8]. At first visit, participant cardiologists gathered detailed information on medical history, laboratory and current drug therapy. Thus, patients underwent baseline echocardiographic study. Patients with abnormal LV wall motion, with reduced LVEF, with heart valve disease defined as more than mild valve regurgitation and/or stenosis of any degree were also rejected. Finally, patients underwent ExSEcho. All patients gave written informed consent and Ethical Committees in the two participating Centers approved the study. The study protocol conforms to the ethical guidelines of the Declaration of Helsinki as revised in 2000.

### Echocardiography

Standard transthoracic Doppler echocardiographic studies were performed using a Megas Esaote Biomedica machine (Florence, Italy) at Trento, and a GE Vivid 9 machine (at Trieste) both equipped with a 2.5–3.5 MHz annular array transducer by two experienced cardiologists (AC, GC) who followed a standardized protocol. Images were stored on CD or MO disks and forwarded for final interpretation at the Echocardiography Core Laboratory at Villa Bianca Hospital of Trento, Italy. Sonographer (GC) was blinded to clinical data. LV chamber dimensions and wall thicknesses were measured by the ASE guidelines [9] and LV mass was calculated using a validated formula [10]. LV mass was normalized for height to the 2.7 power and LV hypertrophy was defined as LV mass > 49.2 g/m ^2.7^ for men and > 46.7 g/m ^2.7^ for women [11]. Relative wall thickness was calculated as the 2* end-diastolic posterior wall thickness/LV diameter ratio and indicated concentric LV geometry if > 0.43 (the 97.5^th^ percentile in a normal population) [12]. LV end-diastolic and end-systolic volumes and stroke volume were measured by the biplane method of disks from 2D apical 4 chamber + 2 chamber views and used to calculate LVEF, defined as reduced if < 50 %. Circumferential end-systolic stress (CESS) was estimated at the LV midwall from M-mode tracings using a cylindric model to assess LV afterload, as previously described [13].

TDI pulsed wave spectral analysis was used to measure peak S’ (mean of 4 measurements obtained in septal, lateral, inferior and anterior mitral annular position), as an estimate of longitudinal LV function [1]. Peak S’ < 8.5 cm/sec (corresponding to the 10^th^ percentile distribution of S’ in the reference healthy population previously analyzed in our center) [5, 6] was considered indicative of longitudinal LVSD.

Transmitral and pulmonary vein pulsed wave Doppler curves and early diastolic TDI velocity of mitral annulus (E’) were assessed according to the ASE recommendations. Early diastolic velocity of transmitral flow (E) was divided by E’ and used to classify LV diastolic function together with other diastolic parameters in 4 degrees as proposed by Redfield et al. [14]: normal, mild dysfunction, moderate dysfunction and severe dysfunction. Maximal left atrial volume was also computed from 2D apical 4-chamber view using the area - length method and was normalized for body surface area. Left atrial systolic force (LASF) was calculated using a validated Manning’s equation [15]. Our group in several papers [16] has reported data on validity, feasibility and reproducibility of LASF.

### Exercise stress echocardiography

All patients underwent symptom-limited ExSEcho (semi-supine position on tilting cycloergometer) with protocol of 25 Watts for 2 minutes workload: heart rate, blood pressure and 12 lead ECG were recorded at rest and every 2 minutes of exercise. Echocardiographic data including LV volumes, LVEF, mitral Doppler and TDI parameters were measured at baseline and at the peak exercise. ExSEcho was considered positive or negative based on symptoms, electrocardiographic and echocardiographic criteria, following the indications of the international guidelines [17]. According to the echocardiographic criteria, ExSEcho was defined positive if wall motion abnormalities developed in at least 1 segment during the stress, while it was defined negative if wall motion did not change [17]. The threshold of the myocardial ischemia was considered low if manifest at 50 watts, high if detected at > 100 watts.

### Statistical analysis

Data are reported as mean values ±1 standard deviation (medians and interquartile ranges for variables deviating from normality) or percentages. Unpaired Student’s test and χ^2^ statistics were used for descriptive statistics. Between-group comparisons of categorical and continuous variables were performed by χ^2^ test and analysis of variance (ANOVA) with comparison between each group by Scheffè test for unequal sample, as appropriate. The study population was stratified in patients with or without inducible myocardial ischemia. A multivariable logistic regression analysis was performed to identify the variables measured at baseline evaluation independently related to the development of myocardial ischemia during ExSEcho. Variables (systolic blood pressure, peak S’, CESS, LASF) significantly related to inducible myocardial ischemia at univariate tests (*p* ≤ 0.01) were included in the multivariate model; age and gender were forced into the statistical model. Receiver operating characteristic (ROC) curve analyses were performed to identify the best cut-off points as the best values for each variable independently associated with the development of inducible myocardial ischemia. To build a predictive score for inducible myocardial ischemia, point = 1 was assigned for each variable outside the predictive cut-off value. The score ranged from 0 (very low risk) to 3 points (very high risk) for inducible myocardial ischemia. All analyses were performed using statistical package SPSS 19.0 (SPSS Inc. Chicago. Illinois) and statistical significance was identified by two-tailed *p* <0.05.

### Reproducibility and feasibility of peak S’

Echocardiographic reproducibility of peak S’ was tested on 50 patients of the study cohort and randomly selected. An expert cardiologist skilled in echocardiography (GC) twice per each patient analyzed data at baseline, 50 Watts and peak exercise. The mean difference between two measurements was ± 4 %. SD of this difference was ± 2 %. Bland-Altman plot showed that in none of these 50 patients the deviation of values of peak S’ measured twice at two different times exceeded the two SD of the mean of peak S’ between the two measures. Inter-observer variability for peak S’ was tested by comparing these measures with those acquired by a second sonographer: the mean difference between two measurements was ± 5 %. SD of this difference was ± 3 %. Intra and inter-observer variability was excellent and did not significantly change when peak S’ was measured at baseline and at peak exercise. Similar data resulted by the comparison between the two different echo machines used for this study (Megas Esaote Biomedica and GE Vivid 9 models). Regarding to the feasibility of peak S’ assessment, no patient had inadequate image quality and the variable could be assessed in all participants.

## Results

### Study population

The baseline clinical and echocardiographic characteristics of the 201 participants are shown in the Tables 1 and 2, respectively. Mean age of the study patients was 65 ± 10 years, 32 % were female, near half of them was affected by hypertension and/or dyslipidemia and/or diabetes. In 87 patients (43 %) the risk SCORE was estimated to be very high. At enrollment, patients were receiving ACE inhibitors and/or angiotensin receptor blockers (ACE/ARB) in the half of cases and beta-blockers in about a quarter of cases. LV hypertrophy was diagnosed in 16 % of the participants, LVSD measured as impaired S’ was detected in one-fifth and LV diastolic dysfunction of any degree in about one fourth of them.

**Table 1:** Main clinical characteristics of the 201 study patients, and comparison between the two study sub-groups classified according to the evidence of myocardial ischemia at the echo exercise test

Variables	NO	YES	*p*	Total study population (201 patients)
	Myocardial ischemia (168 patients, 84 %)	Myocardial ischemia (33 patients, 16 %)		
Age (years)	65 ± 9	66 ± 9	0.50	65 ± 10
Female gender (%)	35	20	0.07	32
Obesity (%)	28	15	0.12	26
Waist circumference (cm)	94 ± 11	93 ± 11	0.16	94 ± 11
Hypertension (%)	46	42	0.72	45
Dyslipidemia (%)	49	45	0.68	48
Active smoker, %	12	12	0.97	12
Diabetes (%)	46	52	0.55	47
Diabetes + hypertension (%)	35	30	0.64	34
Systolic Blood Pressure (mmHg)	140 ± 18	147 ± 23	0.01	141 ± 19
Diastolic Blood Pressure (mmHg)	80 ± 9	81 ± 11	0.35	80 ± 9
Heart Rate (beats/minute)	70 ± 11	67 ± 12	0.11	69 ± 11
Glycemia (mg/dl)	134 ± 59	131 ± 53	0.89	133 ± 58
HbA1c (%) ^a^	7.6 ± 1.4	7.5 ± 1.5	0.88	7.6 ± 1.5
Hemoglobin (gr/dl)	14.3 ± 1.5	14.5 ± 1.5	0.73	14.3 ± 1.5
GFR (ml/min/1.73 m^2^)	81 ± 8	74 ± 10	0.78	80 ± 24
LDL Cholesterol (mg/dl)	110 [92–132]	95 [81–130]	0.75	106 [83–129]
Triglycerides (mg/dl)	162 [118–218]	145 [89–200]	0.10	157 [99–192]
Macroalbuminuria (>300 mg/g) (%)	22	11	0.47	20
Pharmacological treatment				
Betablockers (%)	21	39	0.02	24
ACEi / ARB (%)	46	42	0.67	46
Diuretics (%)	17	18	0.90	17
Calcium antagonists (%)	17	3	0.04	15
Anti-hypertension medications ^b^	1.1 ± 1.1	1.1 ± 1.2	0.95	1.1 ± 1.1
Anti-platelets agents (%)	38	58	0.04	41
Statins (%)	40	45	0.60	41
Metformin (%) ^a^	40	39	0.94	40
Other oral anti-diabetic drugs (%)	20	20	0.97	20
Insulin (%)	16	12	0.70	16

*ACEi* Angiotensin-converting enzyme inhibitors, *ARB* Angiotensin T1 receptor blockers, *GFR* Glomerular Filtration Rate, *HbA1c* glycated haemoglobin

^a^ Measured in patients with diabetes mellitus only; ^b^ number per patient

**Table 2:** Echocardiographic features

Variables	NO	YES	*p*	Total study population (201 patients)
	Myocardial ischemia (168 patients)	Myocardial ischemia (33 patients)		
LV EDD (ml/m^2^)	2.6 ± 0.3	2.7 ± 0.3	0.02	2.6 ± 0.3
LV ESD (ml/m ^2^)	1.6 ± 0.3	1.6 ± 0.4	0.46	1.6 ± 0.3
LV EDV (ml/m^2^)	49 ± 12	51 ± 13	0.45	49 ± 13
LV ESV (ml/m^2^)	18 ± 5	21 ± 9	0.02	19 ± 6
Relative wall thickness	0.39 ± 0.06	0.36 ± 0.07	0.009	0.38 ± 0.06
Concentric LV geometry (%)	21	10	0.12	19
LV mass index (g/m ^2.7^)	42 ± 11	40 ± 7	0.40	41 ± 10
LV hypertrophy (%)	16	15	0.89	16
LV stroke volume (ml)	59 ± 19	57 ± 15	0.60	59 ± 18
LV ejection fraction (%)	63 ± 7	60 ± 9	0.06	62 ± 7
LV CESS (dynes/cm^2^)	120 ± 34	150 ± 53	< 0.001	125 ± 40
Peak S’ (cm/sec)	9.7 ± 1.7	8.7 ± 1.7	0.001	9.6 ± 1.7
Impaired S’ (%)	17	36	0.009	20
Peak E’ (cm/sec)	11.5 ± 2.6	10.8 ± 2.7	0.19	11.4 ± 2.6
E wave of transmitral flow (cm/sec)	70 ± 17	72 ± 23	0.44	70 ± 18
A wave of transmitral flow (cm/sec)	74 ± 18	77 ± 17	0.28	74 ± 18
E / A ratio	0.98 ± 0.29	0.95 ± 0.30	0.60	0.88 ± 0.23
E / E’ ratio	6.4 ± 2.3	7.0 ± 2.7	0.14	6.5 ± 2.3
LV diastolic dysfunction (%)	23	27	0.75	23
Grade I	19	18		35
Grade II	4	9		4
Grade III	0	0		0
Maximal left atrial volume (ml/ m^2^)	23.4 ± 8.4	22.9 ± 9.0	0.60	23.2 ± 8.6
Left atrial systolic force (Kdynes)	14.5 ± 7.9	17.8 ± 9.7	0.01	15.0 ± 8.3

*CESS* circumferential end-systolic stress, *EDD* end-diastolic diameter, *EDV* end-diastolic volume, *ESD* end-systolic diameter, *ESV* end-systolic volume, *LV* left ventricular, *Peak E’* early diastolic Tissue Doppler velocity of mitral annulus, *Peak S’* peak mitral annular systolic velocity (Tissue Doppler Imaging), *Sc* stress corrected

### Exercise stress echocardiography and ischemic patients

The mean duration of ExSEcho in the total study population was 8’55” corresponding to a mean workload of 111 ± 40 watts. According to the results of ExSEcho, patients were divided into two groups: 168 (84 %) non-ischemic and 33 (16 %) ischemic patients. During ExSEcho, among the 33 patients belonging to the ischemic group, 13 (39 %) suffered from typical chest pain, 24 (73 %) had typical electrocardiographic ischemic changes, 28 (85 %) developed changes in LV wall motion (ipokinesia or akinesia in 17 patients in the territory of the right coronary artery and 11 in the left coronary artery). Inducible myocardial ischemia was diagnosed after a mean time from the beginning of exercise of 6’50” corresponding to a mean workload of 84 ± 30 watts. The threshold of the myocardial ischemia was low in 10 patients (30 %), high in 7 patients (21 %), and intermediate in the remaining 16 patients. The baseline clinical and laboratory characteristics of ischemic and non-ischemic patients were similar, but systolic blood pressure was significantly higher in the former, who were treated more frequently with beta-blockers and antiplatelet agents, but less frequently with calcium channel blockers than the latter (Table 1). At the baseline echocardiography, ischemic patients had slightly larger LV size, lower relative wall thickness and higher CESS than non-ischemic patients (Table 2). In the ischemic group, the peak S’ was significantly lower and the longitudinal LV function was impaired more frequently (about two-fold) than in the group of non-ischemic patients. There was no difference in the prevalence of diastolic dysfunction between the two study groups, but LASF was significantly higher in the group of ischemic patients (Table 2).

### Angiographic data

Invasive coronary angiography was proposed in all 33 ischemic patients and performed in 32 of them (one patients refused the invasive diagnostic test). Three patients (9 %) had no or minor lesions (less than 30 % diameter stenosis), 2 (6 %) had intermediate lesions (with 30–69 % diameter stenosis) and 27 (84 %) had significant coronary lesions (≥70 % diameter stenosis). Among these 27 patients, 20 (74 %) underwent percutaneous transcateter coronary angioplasty + stent implantation and 7 (26 %) underwent elective coronary artery by-pass graft surgery.

### Predictors of inducible myocardial ischemia

The variables measured at baseline evaluation associated with the inducible myocardial ischemia at univariate analysis were higher systolic blood pressure, larger LV end-diastolic diameter and end-systolic volume, lower LV relative wall thickness, higher CESS, lower peak S’ and higher LASF. At multivariate logistic regression analysis, higher CESS, lower peak S’ and higher LASF emerged as the independent conditions associated with inducible ischemia in the study population (Table 3).

**Table 3:** Multivariate logistic regression model for prediction of LV myocardial ischemia induced by echo exercise stress

Variables	Adjusted OR	95 % CI	*p*
Peak S’ (decrease of 1 cm/sec)	0.71	0.54–0.93	0.01
LV Circumferential End-Systolic Stress (increase 1 dynes/cm^2^)	1.01	1.00–1.02	0.03
Left atrial systolic force (increase of 1 Kdynes)	1.05	1.01–1.11	0.04
Systolic blood pressure (increase of 1 mmHg)	1.01	0.99–1.04	0.34
Age (years)	1.00	0.95–1.05	0.92
Gender (female)	0.44	0.15–1.33	0.14

Sample size: *n* = 201

*CI* Confidence Intervals, *LV* left Ventricular, *OR* Odds Ratio, *Peak S’* peak mitral annular systolic velocity (Tissue Doppler Imaging), index of LV systolic longitudinal function

### Echo score for prediction of inducible ischemia

**Fig. 1:**
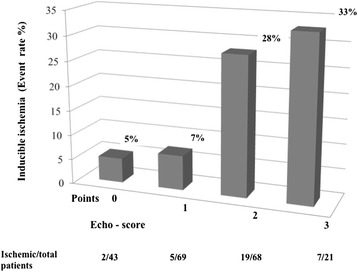
The observed rate of inducible myocardial ischemia in the study patients classified according to all possible values derived by the predictive echo-score. The event rate was 5 % in the patients at lowest risk (score 0) and 33 % in the group at highest risk (score 3)

According to the results of the multivariate regression analysis, a simple echo score was generated after the categorization of the 3 echocardiographic predictors of inducible myocardial ischemia (see “statistical analysis paragraph”) as follows: peak S’ (if <10.0 cm/sec = 1 point, if ≥ 10.0 cm/sec = 0 point); LASF (if >14.0 Kdynes = 1 point, if ≤ 14.0 Kdynes = 0 point); CESS (if >124.0 = 1 point, if ≤ 124.0 dynes/cm^2^ = 0 point). The identification of the best cut-off points as the best values for each variable independently associated with the development of inducible myocardial ischemia was executed by ROC curve analyses and the results are shown in the Table 4. The observed event rate (inducible myocardial ischemia) in the study cohort varied from 5 % to 33 % according to all possible values derived by the predictive score, which ranged from 0 to 3 (Fig. 1).

**Table 4:** Categorization and identification of the best cut-off points of each variable independently associated with the development of inducible myocardial ischemia: the ROC curve analyses

Variables	Cut-off point	AUC [95 % CI]	*Sensitivity*	*Specificity*
Peak S’ (cm/sec)	10.0	0.69 [0.60–0.78]	72 %	60 %
LV CESS (Kdynes/cm^2^)	124.0	0.67 [0.56–0.78]	63 %	60 %
LASF (Kdynes)	14.0	0.61 [0.51–0.72]	63 %	60 %

*AUC* Area Under the Curve, *CESS* Circumferential End-Systolic Stress, *CI* Confidence Intervals, *LASF* Left Atrial Systolic Force, *LV* left ventricular, *Peak S’* peak mitral annular systolic velocity (Tissue Doppler Imaging), index of LV systolic longitudinal function

### Progression of peak S’ and LASF during exercise

**Fig. 2:**
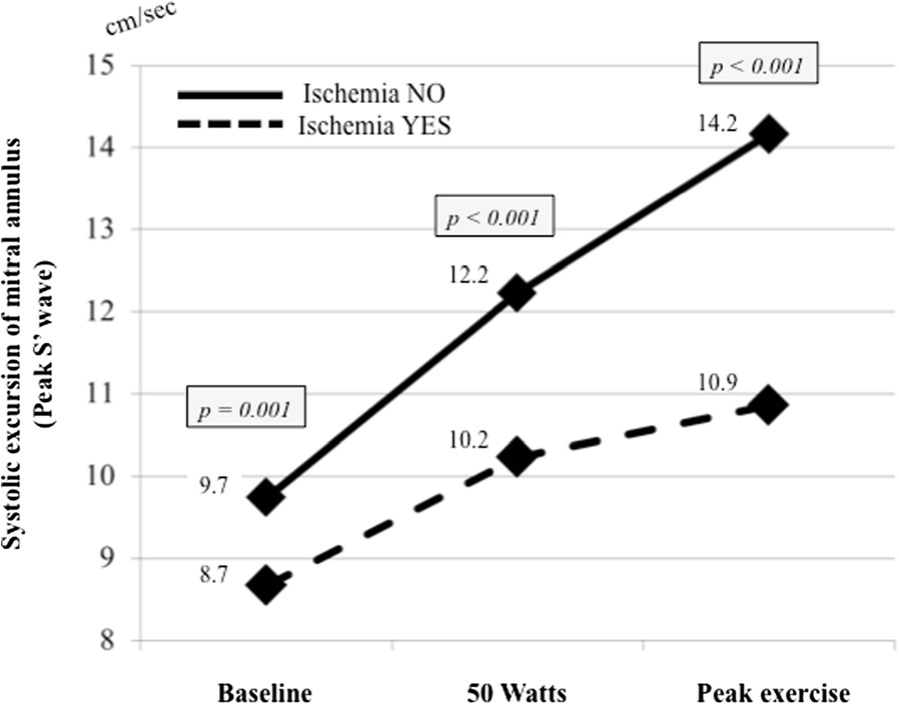
Baseline values and progression during exercise stress echocardiography of peak mitral annular systolic velocity (peak S’) in patients with and without inducible myocardial ischemia

**Fig. 3:**
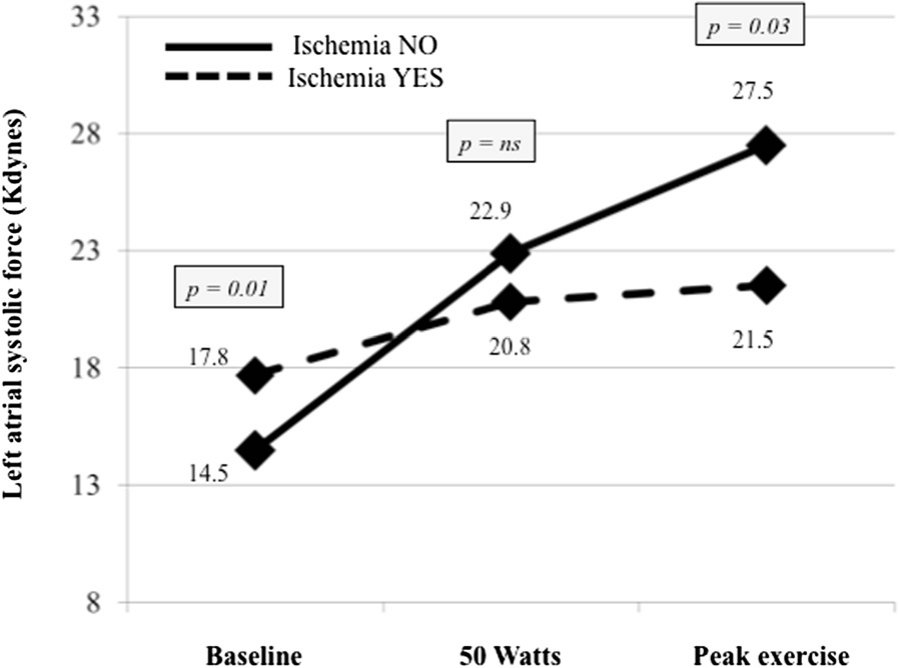
Baseline values and progression during exercise stress echocardiography of left atrial systolic force (LASF) in patients with and without inducible myocardial ischemia

During ExSEcho, the peak S’ remained constantly reduced in the ischemic patients in comparison with the non-ischemic ones: the increase in peak S’ was 25 % in ischemic and 46 % in non-ischemic patients (*p* < 0.001) (Fig. 2). Further, LASF was significantly higher at baseline evaluation in ischemic than non-ischemic patients, hence it increased much less in the former than in the latter during exercise, therefore it was significantly lower in the ischemic than non-ischemic group at the peak exercise (Fig. 3).

## Discussion

The main result emerged by the present study was that in patients at increased risk for CV events, without history of cardiac disease, a reduced longitudinal LV systolic function measured at baseline evaluation was closely associated with inducible myocardial ischemia during ExSEcho, independent of the traditional confounding factors. Furthermore, the coexistence of high CESS and high LASF with a reduced longitudinal LV systolic function predicted the development of inducible myocardial ischemia during ExSEcho in a substantial portion (one third) of the ischemic patients. This is what does our study finding add beyond current day ExSEcho. Unexpectedly, no clinical or laboratory variable including diabetes mellitus, hypertension or renal dysfunction was associated with inducible ischemia in our population.

Peak S’ reduction was already identified as an early marker of longitudinal LV systolic dysfunction in different conditions [2]. Several authors found in diabetic patients a 10–20 % reduction of peak S’ [18, 19] evident either at rest or during ExSEcho in comparison with controls [18, 20]. Similarly, 10 % of asymptomatic hypertensive patients showed a reduction of peak S’, closely correlated with LV hypertrophy and LVSD [21, 22]. Also in patients with coronary artery disease (CAD), peak S’ at rest was reduced [23, 24], and the reduction was positively correlated with the severity of CAD [24]. In other studies [23, 25], significant CAD was associated with higher LV mass, and with several parameters of diastolic and systolic dysfunction. Our results are partially in line with the previous ones: in our population, indeed, TDI identified the presence of inducible myocardial ischemia by the lower values of peak S’ in ischemic patients than controls at rest, but all other Doppler parameters as well as LV mass were not significantly different between the ischemic and non-ischemic groups. Collectively, our findings clearly indicate that patients who developed myocardial ischemia during ExSEcho have an intrinsic and specific LVSD at rest detectable by measuring the global longitudinal function using TDI echocardiography. Furthermore, during ExSEcho, the longitudinal LV function expressed as peak S’ increased significantly less in ischemic than in non-ischemic ones. One reason could be that we induced mainly subendocardial ischemia during ExSEcho and at endocardial layer the longitudinal component of the myocardial fibers shortening is prevalent. Thus, it is not surprising the association between subendocardial ischemia and reduced increase in longitudinal LV systolic function. Evidently, both conditions and their close relationship also exist at rest, although overt signs/symptoms of subendocardial ischemia are lacking.

In our study population, diabetes, hypertension and obesity were widely present. Thus, as predictable, these pathological conditions could not emerge as prognosticators of ExSEcho-induced myocardial ischemia in this setting of patients. No difference, indeed, existed in clinical characteristics between the two study groups. Some difference in drug therapy was present at baseline evaluation but no medication significantly influenced the development of ischemia during ExSEcho.

Together with longitudinal LVSD, another condition independently associated with the inducible myocardial ischemia was the higher LASF. With increased LV stiffness and reduced LV compliance, LASF increases to preserve LV filling and has to be considered as a sensible marker of LV diastolic function [25, 26]. Furthermore, increased LASF is a prognosticator of a higher CV risk in patients with chronic heart failure with preserved LVEF [16], hypertension [27] and aortic stenosis [28]. Our findings may be interpreted as the expression of the inter-dependence between LV systolic and diastolic function, two active processes requiring energy closely coupled in the cardiac cycle which exert a mutual negative influence in presence of excessive LV mass growth and/or myocardial ischemia [29]. Interestingly, LASF increased much less in ischemic than non-ischemic patients during the intermediate phase of ExSEcho and it was significantly lower in the former than in the latter at the peak exercise. Two reasons may justify this behavior recognized by our analysis: 1) the development of atrial ischemia leading to a reduction of systolic atrial performance in ischemic patients 2) the significant increase in LV filling pressures during exercise in ischemic patients leading to a reduction of the peak velocity of the blood at atrial contraction during late diastole despite a preserved atrial systolic force: this condition actualizes a reduction of A wave of the trans-mitral flow and consequently, a reduction of LASF values.

Also an increased LV CESS, row index of LV afterload, was independently associated with inducible myocardial ischemia in our patients. CESS was studied in patients at high CV risk, where it was found increased in presence of hypertension and LV hypertrophy [30]. In our study a new relation between increased CESS at basal echocardiography and inducible myocardial ischemia was found, suggesting higher levels of LV afterload a derivative hemodynamic status of myocardial ischemia or, alternatively, a condition inducing myocardial ischemia. The available data do not allow us to definitely assess the real pathophysiological pathway, so that we can only make speculative inferences about this finding.

Taking one by one the three echocardiographic parameters emerged at multivariate regression analysis as prognosticators of inducible myocardial ischemia (peak S’, LASF and CESS), the sensibility and specificity for the event prediction was around 60 %, slightly better for peak S’. Furthermore, there is significant overlap between the S’ values between the two study groups, resulting in a relatively high number of both false positive and false negative results where S’ value would be considered as continuous variable. But combining the three variables together, on the basis of the cut-off got from ROC analysis, we could better predict inducible myocardial ischemia during ExSEcho at baseline echocardiography. The echocardiographic methods for the assessment of these variables are easy and feasible using standard echocardiography, so that the proposed evaluation can be performed in every echo-Lab without high technology.

### Limitations and strengths of the study

Lacking follow-up data, no prognostic inference in regards to the detection of longitudinal LVSD in patients candidate to ExSEcho can be made. Although our statistical models were extensive, some confounders explaining the observed relations could be left out. The tissue Doppler S’ is an angle dependent measure, so that we cannot exclude that this aspect may have influenced at least in part our results. Finally, the analyses did not consider any parameter of vascular function. Strengths of our study include the large number of participants prospectively enrolled, the reliable, appropriate and validated methods for the assessment of longitudinal LVSD, LASF and CESS as well as the other numerous echocardiographic variables considered in this investigation, the comprehensive nature of the dataset and the capability to correct for the most clinically relevant CV risk factors.

## Conclusions

In asymptomatic patients at increased risk for adverse CV events baseline longitudinal LVSD together with higher LV circumferential end-systolic stress and LASF are the factors associated with myocardial ischemia induced by ExSEcho. The assessment of these factors at standard echocardiography might help the physicians for improving the risk stratification among these patients for ExSEcho. This approach could lead to save substantial human and economic resources. The present findings might have an extensive clinical applicability mostly because ExSEcho has the advantages of its wide availability, safety, low cost and versatility for the assessment of inducible myocardial ischemia.

## Abbreviations

ACE, Angiotensin-converting enzyme; ARB, Angiotensin receptor blockers; ASE, American Society of Echocardiography; CAD, Coronary artery disease; CESS, Circumferential end-systolic stress; CV, Cardiovascular; ExSEcho, Exercise stress echocardiography; LASF, Left atrial systolic force; LV, Left ventricular; LVEF, Left ventricular ejection fraction; LVSD, Left ventricular systolic dysfunction; ROC, Receiver operating characteristic; S’, Mitral annular peak systolic velocity; TDI, Tissue Doppler Imaging.
